# Poisoning by Corrosive Chloride of Mercury

**Published:** 1857-08

**Authors:** 


IOWA MEDICAL JOURNAL.

J. C. HUGHES, } EDITORS. { WELLS R. MARSH,

VOL. IV. KEOKUK, IOWA, JULY AND AUGUST, 1857. NO. 2.

### ORIGINAL COMMUNICATIONS.

### Poisoning by Corrosive Chloride of Mercury.

[Case Communicated for Iowa Medical Journal.]

Margaret ——— attempted to poison herself by taking laudanum in the evening; failing in the attempt because of the occurrence of vomiting soon after its ingestion, she on the following morning essayed to accomplish her purpose with "bed-bug poison." About 11 o'clock, A. M. I was called to see her in consultation with Dr. Ladd, who had just been called to see the case, but had as yet done nothing for her.

Found the patient sitting up. The surface bathed in profuse cold sweat—pulse over a hundred and forty, and small—pupils contracted—countenance anxious—respiration rapid. Complains of pain in the fauces, epigastrium and abdomen. Has vomited repeatedly, but only a small quantity of bloody mucus has been ejected each time, together with a little water which she had from time to time drank. Has had frequent stools but is unable to give farther information as to their character, than that they have been copious and thin. Says she cannot swallow even cold water any more.

Upon first seeing the patient, and previous to a full examination and inquiry, I was led by her general appearance to suspect her of taking opium, and charged her with it. She said she had taken laudanum the night before, but vomited it up; said, when questioned as to the quantity, that she had taken it from a bottle kept by the family and had taken all there was in it. Upon obtaining the bottle and questioning the family, found that she must have taken from an ounce and a half to two ounces of the officinal tincture, but had suffered no further inconvenience from it than the emesis, and a restless and sleepless night.

While the family were at breakfast at about seven o'clock, she had had resort to the "bed-bug poison," taking, as she says "two great swallows," and experienced for half an hour only a violent burning pain in the mouth, throat and stomach. From a half to three-quarters of an hour after the ingestion of the drug, she commenced vomiting and purging, as before remarked; and these symptoms, together with the pain, had continued to the present time—four hours —when the patient was in the condition above described.

Prescribed free administration of the whites of eggs, but as these were not at hand, administered, or attempted to administer, an emulsion of wheat flour. Patient refuses to take it upon the plea of inability to swallow. Procured a stomach-pump and threw into the stomach about a pint of the emulsion, and reversing the connection of the pump, evacuated the stomach again and then administered as much more. After a short interval evacuated the stomach again by means of the pump, and the eggs having arrived, administered the whites of eight, and again withdrew the pump tube.

From this time, I may remark, the patient found it more pleasant to recover and exercise the power of deglutition, than submit to the use of the pump. Patient is to have the whites of two eggs every half hour. Dry heat to extremities; Discharges from the bowels every few minutes; are copious, and consist almost exclusively of partially coagulated blood. Complains of great thirst, but chooses, because of painful deglutition, to suffer the thirst rather than to drink save when the antidote is ordered.

1 1-2 o'clock, P. M. Pulse, 148; discharges from bowels still continue, but contain, along with the blood, some fecal matter and considerable bile. Skin warmer, less livid, and perspiration less profuse. Continue the treatment.

Examined by tests some of the matter ejected from the stomach, finding the corrosive chloride of mercury largely present.

3 o'clock, P. M. Patient improved in general appearance; pulse 140 and somewhat more full; discharges from the bowels less frequent, but still copious and of the same character as at last visit. Extreme tenderness over whole abdomen. Salivary secretion profuse; but no redness of the gums, and the fauces and buccal membrane are but slightly reddened. Continue treatment; warm fomentations to abdomen.

7 o'clock, P. M. Still improving; pulse 132 and steadier, with better volume. Skin and extremities warm; has had but two stools since last visit. Less pain—less thirst. Countenance much better.

10 o'clock, P. M. General improvement continues, but flow of saliva increased.

Second day, 7 o'clock, A. M. Margaret is much better. Has had one evacuation from bowels this morning which contains only a small amount of blood, and that has no longer the fresh appearance of yesterday. Pulse 115, and soft; Skin natural, or nearly so. Abdominal tenderness subsiding

Continue fomentations and let the patient have mucilaginous drinks, and gargle the mouth with a solution of chlorate of potash.

From this time forward, the patient rapidly convalesced, the salivation gradually subsided, the abdominal tenderness gave way and on the seventh day no evidence of the effects of poison remained save slight debility.

This case is noteworthy from the slight toxic effect of so large a quantity of the drug so long retained. The solution swallowed was made by using an ounce of the corrosive sublimate to a pint of dilute alcohol and the solution was complete. She could not have taken less than from an ounce to an ounce and a half of the solution which contained a half drachm to the fluid ounce. This amount, (thirty to forty-five grains,) in a condition most favorable for the production of poisonous influence, was taken upon an empty stomach and retained at least half an hour before emesis occurred at all, and yet the matter vomited evinced but slight erosion of the gastric mucus surface. The greatest irritant effect was experienced below the stomach in both the small and large intestines. The discharges from the bowels were not tested for the presence of the poison; but, as before remarked, it was found to be still present in the stomach five or six hours after its ingestion, and that too in no small quantity.

The large quantity of blood discharged by the bowels evinces the amount of erosion of the intestinal canal, but after the third day all inflammatory symptoms had disappeared; and the salivation, profuse on the evening after the poisoning, had subsided entirely on the morning of the fifth day.

Keokuk, July 1st, 1857.

